# A Novel Erythromycin Resistance Plasmid from *Bacillus* Sp. Strain HS24, Isolated from the Marine Sponge *Haliclona Simulans*

**DOI:** 10.1371/journal.pone.0115583

**Published:** 2014-12-30

**Authors:** Teresa M. Barbosa, Robert W. Phelan, Dara Leong, John P. Morrissey, Claire Adams, Alan D. W. Dobson, Fergal O’Gara

**Affiliations:** 1 School of Pharmacy, University College Cork, Cork, Ireland; 2 Department of Microbiology, University College Cork, Cork, Ireland; 3 Biomerit Research Centre, University College Cork, Cork, Ireland; 4 Marine Biotechnology Centre, Environmental Research Institute, University College Cork, Cork, Ireland; 5 Curtin University, School of Biomedical Sciences, Perth WA 6845, Australia; Catalan Institute for Water Research (ICRA), Spain

## Abstract

A better understanding of the origin and natural reservoirs of resistance determinants is fundamental to efficiently tackle antibiotic resistance. This paper reports the identification of a novel 5.8 kb erythromycin resistance plasmid, from *Bacillus* sp. HS24 isolated from the marine sponge *Haliclona simulans.* pBHS24B has a mosaic structure and carries the erythromycin resistance gene *erm*(T). This is the first report of an erythromycin resistance plasmid from a sponge associated bacteria and of the Erm(T) determinant in the genus *Bacillus*.

## Introduction

Antibiotic resistance is recognised as a major public health problem and resistance determinants have been identified in a wide variety of different clinical and environmental settings [1], [2], [3], [4], [5]. However, despite many years of research, the origin of these resistance determinants remains elusive [6], [7]. Resistance genes are frequently associated with promiscuous mobile genetic elements which drive their evolution and facilitate their horizontal spread [8]. Knowledge on the prevalence and nature of these in natural habitats is therefore fundamental to increasing our understanding of the development of antibiotic resistance [9]. Additionally, these plasmids can provide a backbone for the creation of new cloning vectors for use in the genetic manipulation of natural isolates, which are frequently refractory to the uptake and integration of exogenous DNA [10].

While the marine sponge microbiota is attracting increasing interest, research to date has primarily focused on the overall microbial diversity and biotechnological potential of this unique microbial ecosystem [11], [12], [13], [14]. However the antimicrobial susceptibility of the sponge microbiota coupled with their ability to act as a possible reservoir for antibiotic resistance determinants; potentially transmissible to the food chain and clinical relevant bacteria [5], has not to date been adequately examined [15], [16].

We have recently isolated a *Bacillus* sp. isolate, HS24, from the marine sponge *Haliclona simulans* [17], [18]. *Bacillus* sp. HS24 displays resistance towards erythromycin and tetracycline and was shown to contain two small plasmids, of which, pBHS24 carries the tetracycline resistance determinant Tet(L) [17]. pBHS24 was shown to be almost identical to three other mobilisable tetracycline resistance plasmids identified in the honey bee pathogen *Paenibacillus larvae* (pMA67), in the anaerobe *Lactobacillus sakei* Rits 9, isolated from an Italian Sola cheese (pLS55) and in the spore-former *Sporosarcina ureae* (pSU1), isolated from the subsurface beneath a broiler chicken farm [17].

In this background, the aim of the present study was to characterise the nature of erythromycin resistance of a halophilic *Bacillus* strain isolated from the marine sponge *Haliclona simulans*.

## Materials and Methods

### Growth and antibiotic susceptibility testing

Sponge-associated *Bacillus* sp. HS24 was routinely grown and maintained aerobically, on Difco marine agar/broth (MA/MB) (Difco 2216), at 30°C, unless otherwise stated. Luria-Bertani medium was routinely used for growth and maintenance of *E. coli* and *B. subtilis* 168.

Susceptibility to erythromycin was determined by spotting MB cultures onto Muller–Hinton (MH, Merck, Darmstadt, Germany) plates supplemented with different concentrations of erythromycin (Sigma-Aldrich, Munich, Germany) and incubated aerobically at 30°C. Initial tests were performed with plates supplemented with 0 to 0.5 mg ml^−1^ erythromycin. The concentration range of erythromycin was subsequently expanded, with plates supplemented with 1.0, 1.5, 2.0, 2.5, 3.0 and 3.5 mg ml^−1^. MIC values are defined as the minimal concentration of antibiotic able to inhibit the growth. As there are no specific established antibiotic breakpoints values for marine sponge *Bacillus* isolates, the breakpoint values used for categorizing strain HS24 as resistant were those recommended by EFSA [19].

### DNA extraction, PCR amplification and transformation

Total genomic DNA of *Bacillus* sp. HS24 was extracted from 24 h MB cultures as previously described [20]. Total plasmid DNA was extracted from overnight MB cultures using the QIAprep Spin miniprep kit optimized for *Bacillus* (Qiagen GmbH, Hilden, Germany).

Plasmid DNA isolated from isolate HS24 was used to transform *B. subtilis* 168 competent cells as previously described [21], [22].

The universal eubacterial primers 27f (5′-AGA GTT TGA TCM TGG CTC AG-3′, M = C or A) and 1492r (5′-GGT TAC CTT GTT ACG ACT T-3′) [23] were used to amplify the small-subunit rRNA (16S rRNA) gene sequence of *Bacillus* sp. HS24. PCR mixtures (50 µl) contained 50 ng of genomic DNA as template, 1× BioTaq PCR Buffer (Bioline, London, UK), 1.5 mmol l^−1^ of MgCl_2_, 0.2 mmol l^−1^ of dNTPs, 0.5 µmol l^−1^ of each primer and 2.5 U of BioTaq DNA polymerase (Bioline). PCR was carried out under the following cycling conditions: initial denaturation at 94°C for 5 min, followed by 30 cycles of 94°C for 30 s, 52°C for 30 s and 72°C for 45 s, with a final extension at 72°C for 10 min.

### DNA sequencing

The near complete 16S rRNA gene sequence of *Bacillus* sp. HS24 (1441 nt) (GenBank JF803858) obtained with the primers 27f and HS24F2 (GTGAAATGCGTAGATATGTGG) (GATC Biotech AG, Germany) was compared with sequences in the Genbank nucleotide sequence database (http://blast.ncbi.nlm.nih.gov/Blast.cgi) using BLASTn [24], [25]. A Neighbour-joining phylogenetic tree was generated by analysing near complete 16S rRNA gene sequences of *Bacillus* sp. HS24 and strains of closely related *Bacillus* species. The tree was constructed using maximum composite likelihood and pairwise deletion. Percentage bootstrap values (>50% only) from 1000 re-samplings are indicated at each node. Bar, 5% estimated sequence divergence.

The pBHS24B plasmid was sequenced as follows: pBHS24B DNA restricted with *Hind*III and *Eco*RI was cloned into the vector pUC18 and initial nucleotide sequences obtained with the M13 primers (GATC Biotech AG, Germany), as previously described for plasmid pBHS24 [17]. The complete nucleotide sequence of the plasmid (GenBank KC991136) was subsequently determined by primer walking, using pBHS24B as a template. The sequencing data was manually assembled using Bioedit [26]. Open reading frames (ORFs) were determined and annotated using ORF Finder (http://www.ncbi.nlm.nih.gov/gorf/gorf.html), and Basic Local Alignment Search Tool (BLAST) at NCBI [27].

### Nucleotide sequence accession numbers

The 16S rRNA gene sequence of *Bacillus* sp. HS24 and the complete nucleotide sequence of plasmid pBHS24B have been deposited in the GenBank database with the accession numbers JF803858 and KC991136, respectively.

## Results and Discussion

**Figure 1:**
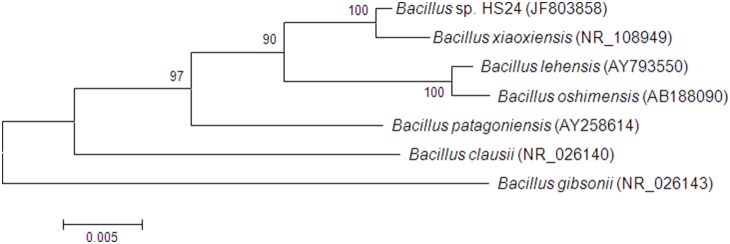
Neighbour-joining phylogenetic tree generated by analysing near complete 16S rRNA gene sequences of *Bacillus* sp. HS24 and strains of closely related *Bacillus* species. Accession numbers are in parentheses. The tree was constructed using maximum composite likelihood and pairwise deletion. Percentage bootstrap values (>50% only) from 1000 re-samplings are indicated at each node. Bar, 5% estimated sequence divergence.

Phylogenetic analysis of the 16S rRNA gene sequence of *Bacillus* sp. HS24 indicates a 99% sequence identity with the 16S rRNA gene of its closest relative, the slightly halophilic *Bacillus xiaoxiensis* strain JSM081004 [28] (Fig. 1).

**Figure 2:**
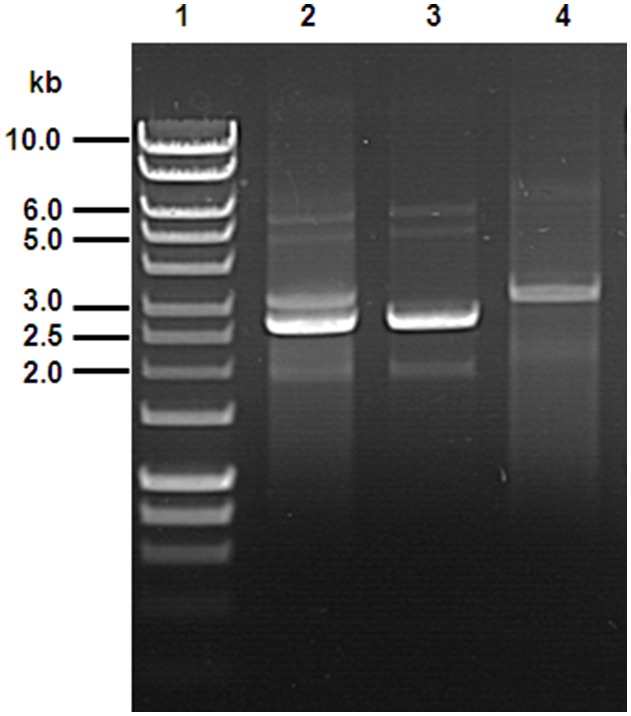
Comparison of plasmid DNA extracted from *Bacillus* sp. strain HS24 and *B. subtilis* 168 transformed with the tetracycline resistance plasmid pBHS24 and the erythromycin resistance plasmid pBHS24B. Lane 1, DNA marker; Lane 2, *Bacillus* sp. HS24; Lane 3, *B. subtilis* 168 - pBHS24; Lane 4, *B. subtilis* 168 - pBHS24B; Multiple faint bands on lanes 2 to 4 correspond to the different conformational forms of plasmid DNA.

Although *Bacillus* sp. HS24 displays high levels of resistance to erythromycin (MIC of 3 mg ml^−1^), transformation of the erythromycin susceptible strain *Bacillus subtilis* 168, with total plasmid DNA purified from isolate HS24, yielded no colonies on LB medium supplemented with 5 µg ml^−1^ erythromycin [17]. However, in the current study a large number of erythromycin resistant transformants were obtained when selection was performed at a lower concentration (1 µg ml^−1^). As expected no colonies were observed on antibiotic control plates when plasmid DNA was not added to cells. A single plasmid of approximately 5.8 kb in size, here named pBHS24B, was purified from the erythromycin resistance *B. subtilis* transformants (Fig. 2). While the level of tetracycline resistance conferred by pBHS24 (>100 µg ml^−1^) in the *B. subtilis* background was significantly higher than that in strain HS24 (75 µg ml^−1^) [17], there was no difference in the level of erythromycin resistance conferred by pBHS24B in the native and cloning hosts. Attempts to transform pBHS24B into chemically competent *E. coli* DH5α or K12 MG1655 cells proved unsuccessful.

**Figure 3:**
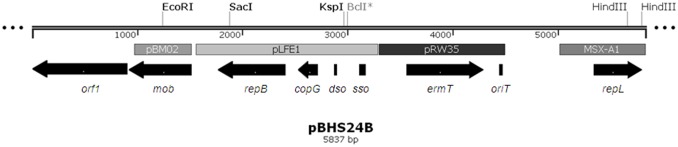
Graphical representation of the genomic structure of pBHS24B from *Bacillus* sp. HS24. Restriction sites and regions with homology to previously reported sequences are indicated. Arrow heads indicate the direction of transcription of the different open reading frames. The 525 bp region immediately upstream from *repL* does not share homology to any other sequence in the database. MSX-A1, *B. cereus* whole genome shotgun (WGS) entry; preliminary data, plasmid content unknown. Figure created using Snapgene viewer.

The pBHS24B sequencing data was manually assembled using Bioedit [26], generating a circular element of 5837 nt (Fig. 3). A total of six putative open reading frames (ORFs) were determined and annotated (Fig. 3, Table 1). Results from BLASTx searches revealed that pBHS24B has a mosaic structure, which is more than likely to have evolved through the occurrence of multiple recombination events in one or more hosts. Different sections of the plasmid appear to have assorted origins as indicated by the level of sequence homology to different extra chromosomal elements from host strains isolated from a wide range of environments and the different G/C content of the respective open reading frames (Table 1, Fig. 3).

**Table 1:** Sequence homology of the proteins encoded by pBHS24B*.

ORF	% G/C content	No. aa^&^	Closest proteinhomologue	Strain/Origin	% aa Identity	E value^#^	Accessio no.
1	44.7	297	–¥	–	–	–	–
2	47	196	Mob like protein,pBM02	*Lactococcus lactis*subsp. *Cremoris* P8-2-47;component of a Germanindustrial starter culture	47	2E-47	NC_004930
3	33.8	212	RepB, pLFE1	*Lactobacillus plantarum*M345; raw-milk cheese	99	5E-157	NC_012628
4	30	59	CopG, pLFE1	*Lactobacillus plantarum*M345; raw-milk cheese	97	8E-22	NC_012628
5	25	244	Erm(T), pRW35	*Streptococcus pyogenes*RW35; nosocomial sample£	100	1E-174	NC_010423
6	36.6	152	Predicted RepL**	*Bacillus cereus* MSX-A1***	83	2E-85	EJQ95744

*Results are from a BLASTx search of the GenBank non-redundant protein database on 13/8/13. ^&^aa, amino acids. ^#^Expectation value.

£ 100% identity also found to other plasmids as described in the text.

**Whole genome shotgun (WGS) entry; preliminary data, plasmid content unknown. ***anthrax-like illness; isolated in Antarctica.

¥ ORF1 shows a low homology hit (27%; E value 3E-05) with a *Leishmania major* structural maintenance of chromosome (SMC) protein domain (CAJ07774).

**Figure 4:**
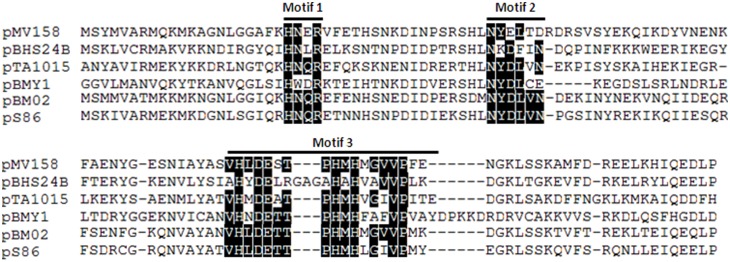
Alignment of pBHS24B and selected pMV158-superfamily relaxases. pS86, *Enterococcus faecalis;* pBM02, *Lactococcus lactis*; pBMY1, *Bacillus mycoides;* pTA1015, *Bacillus subtilis;* pMV158, *Streptococcus agalactiae.* The three conserved motif sequences typical of the pMV158 family of Pre/Mob proteins are identified [30]. Conserved amino acids within the motifs are highlighted in black.

pBHS24B encodes a truncated copy of the recombinase/mobilisation gene, *pre*/*mob*, whose deduced amino acid sequence shows the highest homology (47% amino acid sequence identity) with the N-terminal 186 aa of the Pre/Mob protein from plasmid pBM02 of *Lactococcus lactis* subsp. *cremoris* [29] (Table 1). The shorter size of the pBHS24B Mob protein (196 amino acid) contrasts with the usually larger Pre/Mob proteins of the pMV158 family (350–500 amino acid) [30]. Although this region spans the three conserved motifs of the pMV158 family of Pre/Mob proteins (Fig. 4) it is not clear as yet if the truncated protein is functional. Sequence analysis suggest that a 894 bp segment of unknown origin, which appears to encode a 297 amino acids hypothetical protein (ORF1), might have integrated at this point in the plasmid resulting in the truncation of the original *pre*/*mob* gene (Fig. 3, Table 1).

The putative replication region of pBHS24B is highly homologous to that of the *erm*(B) encoding rolling circle-replication (RCR) plasmid pLFE1, from the raw milk cheese isolate *Lactobacillus plantarium* M345 [31]. This includes the copy number control protein, CopG and the replication initiation protein, RepB (with only one and two nucleotide differences between the *copG* and *repB* genes, respectively, in the two plasmids) (Table 1). This homology also extends to a 580 bp region upstream of *copG*, which includes a putative replication initiation site with a single-strand origin (*sso*)-like region and a characteristic pMV158 family double-strand origin (*dso*) (100% nt sequence identity) [31]. This again suggests that pBHS24B belongs to the pMV158 family of plasmids [30], and therefore is likely to replicate by a RCR mechanism, like many of the plasmids derived from Gram positive hosts [32].

A second putative replication initiation protein with 83% amino acid sequence identity to the putative RepL protein from *Bacillus cereus* MSX-A1 (Genbank accession number EJQ95744) is also present in pBHS24B (Fig. 3, Table 1). Replication proteins of the RepL family are frequently found in small cryptic or erythromycin resistance encoding RCR plasmids previously identified in *Staphylococcus* and *Bacillus* species [33]. The presence of more than one replication protein has previously been reported for other plasmids, such as the *Streptococcus faecalis* plasmid pAMα1 [34] and the *Bacillus* plasmid pTB19 [35], [36].

Erythromycin resistance in pBHS24B is conferred by a macrolide-lincosamide-streptogramin B (MLS_B_) resistance methylase Erm(T), which has been previously reported only in the genera *Enterococcus*, *Lactobacillus*, *Streptococcus* and *Staphylococcus* (http://faculty.washington.edu/marilynr/). The pBHS24B Erm(T) protein shares 100% amino acid sequence homology with the Erm(T) of pUR2940, pUR2941, pKKS25, pRW35, pGA2000, pGB2001 and pGB2002 isolated from *Staphylococcus aureus, Streptococcus agalactiae* and *Streptococcus pyogenes* strains [34], [37], [38], [39], [40] (Table 1). The *oriT* sequence is located downstream of *erm*(T) and should have the same origin as the resistance gene (Fig. 3). The sequences encompassing the leader peptide-encoding sequence and the *erm*(T) translational start regions of these plasmids are also identical [38]. Previous comparisons of the *erm*(T) up- and downstream sequences in the streptococcal pGB2002, pGB2001, pGA2000, pRW35 and the staphylococcal pUR2940, pUR2941 plasmids, identified 56 to 58 bp long conserved imperfect direct repeat (IDR) regions [39], which are believed to play a role in the acquisition of the erythromycin resistance determinants. Although the downstream sequence is clearly identifiable and relatively well conserved in pBHS24B (4437–4492 nt), the acquisition of a 1730 bp fragment of DNA from plasmid pLFE1 (Fig. 3), appears to have resulted in the deletion of the IDR region upstream of *erm*(T). *Bacillus* sp. HS24 has not been screened for other previously described erythromycin resistance determinants, nor has it been cured of plasmid pBHS24B, and therefore, the concomitant existence of other erythromycin resistance gene(s) in the genome of this strain cannot be excluded.

To our knowledge this is the first report of the erythromycin resistance Erm(T) determinant in the genus *Bacillus*. Erythromycin resistance through methylation of the 23S rRNA within this genus, has been previously associated with Erm(B), Erm(C), Erm(D), Erm(G) and Erm(34), with evidence for specific species association for some of the determinants (http://faculty.washington.edu/marilynr/ermweb4.pdf) [41]. The *erm*(T) gene has previously been identified in bacterial isolates from agricultural and clinical settings [42], [43], [44], [45], where the widespread use of antibiotics is likely to have contributed to the development of resistance within the associated microbiota. While the prevalence of erythromycin resistance among marine sponge bacteria is unknown, *B. licheniformis* HS147, was the only other *Bacillus* isolate from *H. simulans* to display resistance to this antibiotic [18]. Antibiotics used in therapy and agriculture are known to accumulate in the environment and to contaminate aquatic habitats where they can exert their selective pressure on the native flora [7], [46], [47]. Erythromycin in particular is widely used to control the spread of infection in the aquaculture industry [48]. Interestingly, the *H. simulans* sponge host of isolate HS24, was recovered from Gurraig Sound in Kilkieran Bay, off the coast of Galway in Ireland [49], in an area that is well known for aquaculture (Status of Irish Aquaculture 2007, http://www.marine.ie/home/Aquaculture.htm). Given that sponges are known to filter large quantities of seawater, up to 24,000 L Kg^−1^ per day; they are thus likely to be susceptible to accumulate environmental contaminants, such as heavy metals and antibiotics, which could ultimately drive the acquisition of resistance by the associated microbiota [15]. Despite fears that intensive aquaculture processes may contribute to the development and dissemination of antibiotic resistance, little is known about this practise in comparison to animal husbandry. The use of antibiotics to treat infection in aquaculture generally focuses on specific fish pathogens and not the complex commensal microbiota of the fish and surrounding marine environments [5].

The mosaic structure of plasmid pBHS24B supports the importance of these elements in the evolution and acquisition of antibiotic resistance through horizontal gene transfer. The question as to whether resistance in this habitat arose as a consequence of environmental contamination or if resistance determinants are a common part of the genome of environmental bacteria where they have alternative functional roles remains highly debatable [9]. Although the overuse and misuse of antibiotics is reported to be responsible for the spread of antibiotic resistant bacteria, a large number of environmental strains produce antibiotics and so potentially carry genes encoding resistance to these compounds. As a result, antibiotics produced in the environment may exert a selective pressure on neighbouring microorganisms [7].

In conclusion *Bacillus* sp. HS24 contains two antibiotic resistance plasmids, one of which is nearly identical to plasmids from commensal and pathogenic bacterial species from four different genera, isolated from quite distinct ecological habitats [17]. The second plasmid shows a mosaic structure, which is likely to have been derived as a result of multiple recombination events between different plasmids within multiple hosts, the order of which remains unknown. Our results further illustrate the promiscuity of the nature of antibiotic resistance and suggest that sponge associated bacteria, as with other environmental bacteria; may represent a reservoir of resistance genes with the potential to transfer resistance to the food chain or indeed clinically relevant organism.
